# Bat Eyes Have Ultraviolet-Sensitive Cone Photoreceptors

**DOI:** 10.1371/journal.pone.0006390

**Published:** 2009-07-28

**Authors:** Brigitte Müller, Martin Glösmann, Leo Peichl, Gabriel C. Knop, Cornelia Hagemann, Josef Ammermüller

**Affiliations:** 1 Max Planck Institute for Brain Research, Frankfurt/Main, Germany; 2 Department of Neurobiology, University of Oldenburg, Oldenburg, Germany; 3 Department of Cell Biology and Neuroscience, University of Frankfurt, Frankfurt/Main, Germany; University of California Davis, United States of America

## Abstract

Mammalian retinae have rod photoreceptors for night vision and cone photoreceptors for daylight and colour vision. For colour discrimination, most mammals possess two cone populations with two visual pigments (opsins) that have absorption maxima at short wavelengths (blue or ultraviolet light) and long wavelengths (green or red light). Microchiropteran bats, which use echolocation to navigate and forage in complete darkness, have long been considered to have pure rod retinae. Here we use opsin immunohistochemistry to show that two phyllostomid microbats, *Glossophaga soricina* and *Carollia perspicillata*, possess a significant population of cones and express two cone opsins, a shortwave-sensitive (S) opsin and a longwave-sensitive (L) opsin. A substantial population of cones expresses S opsin exclusively, whereas the other cones mostly coexpress L and S opsin. S opsin gene analysis suggests ultraviolet (UV, wavelengths <400 nm) sensitivity, and corneal electroretinogram recordings reveal an elevated sensitivity to UV light which is mediated by an S cone visual pigment. Therefore bats have retained the ancestral UV tuning of the S cone pigment. We conclude that bats have the prerequisite for daylight vision, dichromatic colour vision, and UV vision. For bats, the UV-sensitive cones may be advantageous for visual orientation at twilight, predator avoidance, and detection of UV-reflecting flowers for those that feed on nectar.

## Introduction

Cone photoreceptors are used for daylight vision, and most mammals possess two cone populations with two visual pigments (opsins) that have absorption maxima in the short-wavelength (blue or ultraviolet light) and long-wavelength (green or red light) ranges and provide the basis for dichromatic colour discrimination [1], [2]. It is thought that only a few species have retained cone-mediated ultraviolet (UV, wavelengths <400 nm) vision, including some rodents [3], [4], while most diurnal mammals cannot see UV light because the absorption maximum of the ancestral UV-sensitive cone visual pigment shifted to violet/blue during evolution [5], [6]. In addition to the blue-shifted short-wave-sensitive (S) opsins, the potentially damaging daylight UV components are blocked by UV-opaque eye media [7]. The eyes of microchiropteran bats are small and their retinas are rod-dominated. Early anatomical studies concluded that bats completely lack cones [8]. More recently, cone opsins and cones were demonstrated in some microbat species. A molecular study found L and S cone opsin genes in the insect-eating bat *Myotis velifer*, but provided no evidence for their expression in retinal photoreceptors [9]. A histological study of the greater horseshoe bat *Rhinolophus ferrumequinum* reported L cones, but did not assess S opsin expression [10]. On the other hand, a behavioural study of the flower bat *Glossophaga soricina* in dark-adapted conditions found no evidence for colour discrimination, but did detect UV sensitivity and concluded that this was a property of the β-band of the rod opsin, and that *G. soricina* lacked a separate shortwave-sensitive cone photoreceptor [11]. The β-band is a secondary absorption peak in the UV region that is a property of the protein moiety of every visual pigment. The only published electrophysiological study on spectral sensitivity of bat photoreceptors examined four microchiropteran species, including *Carollia perspicillata*; that study postulated the existence of two visual pigments: a rod opsin (λ_max_ 500 nm) and a second pigment absorbing at about 560–580 nm [12]. A UV-sensitive pigment was not addressed in that study because stimuli were limited to wavelengths >440 nm.

The S opsin amino acid sequence of the insect-eating *Myotis velifer* suggests UV tuning, but has not been corroborated physiologically [9]. Therefore, we aimed to assess whether bats have cones, a prerequisite for daylight and colour vision, and whether the cones express different types of opsins. Furthermore, we aimed to demonstrate UV sensitivity by sequencing the tuning-relevant segment of the S opsin gene and by corneal electroretinograms, measuring retinal action spectra S(λ) with and without chromatic adaptation. The results of our study indicate cone-based UV sensitivity in phyllostomid bats.

## Results

### Detection of Rod and Cone Opsins

**Figure 1:**
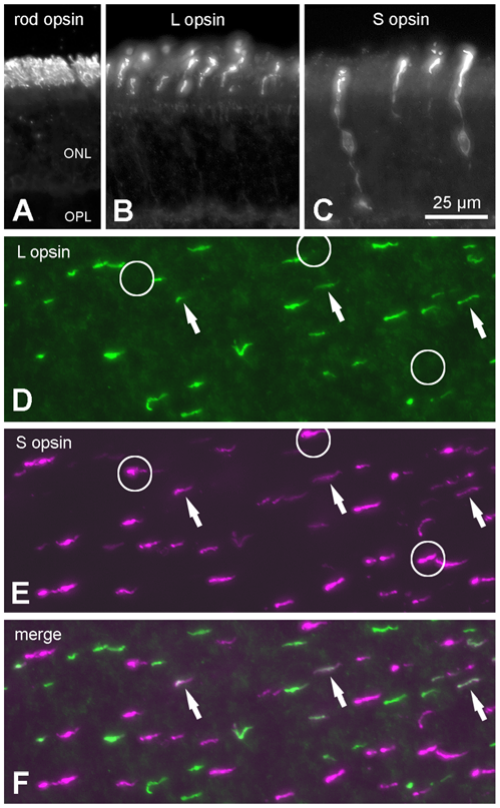
Rod and cone photoreceptors in the retina of *C. perspicillata* and *G. soricina*. (A–C) Vertical sections of *C. perspicillata* retina immunostained for rod opsin (A), long-wave-sensitive (L) opsin (B) and short-wave-sensitive (S) opsin (C). Commonly, the antibodies labelled only the photoreceptor outer segments, but the S opsin antibodies also weakly labelled the somata and axons. (D–F) Double immunofluorescence labelling for the cone opsins in a flat-mounted retina of *G. soricina*. Examples of cones expressing both opsins are indicated by arrows, cones expressing S opsin only by circles. Cone outer segments containing roughly equal amounts of both opsins appear whitish in the merge. All micrographs shown at same magnification. ONL, outer nuclear layer; OPL, outer plexiform layer.

**Figure 2:**
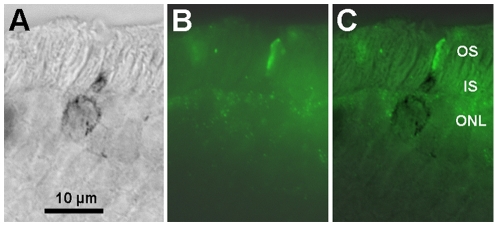
Combination of *in situ* hybridization and immunohistochemistry in a vertical section of *C. perspicillata* retina. (A) Short-wave-sensitive (S) cone opsin transcript in a cone photoreceptor soma and inner segment. (B) Immunolabelling of S opsin in the photoreceptor outer segment. (C) Merging the two labels demonstrates that the transcript and the protein are in the same cell. OS, layer of photoreceptor outer segments; IS, layer of photoreceptor inner segements; ONL, outer nuclear layer.

We used immunocytochemistry with antibodies against mammalian opsins to detect one rod opsin and two cone opsins in the outer segments of separate retinal photoreceptor populations in *Glossophaga soricina* and *Carollia perspicillata* (Fig. 1A–C). Photoreceptors labelled by antibodies against the short-wave-sensitive (S) and long-wave-sensitive (L) cone opsins were also labelled by the general cone marker peanut agglutinin and comprised 2–4% of all photoreceptors (not shown). Almost every L cone also expressed some amount of S opsin, whereas a considerable population of genuine S cones expressed S opsin exclusively. Cones expressing both L and S opsins (dual pigment cones, Fig. 1D–F) were present at very high proportions, locally reaching up to 100% of the cones. Using *in situ* hybridisation in *C. perspicillata*, we detected L and S cone opsin transcripts in a subset of photoreceptor somata. By combining *in situ* hybridisation with immunocytochemistry, we established that the respective cone visual pigment mRNA was translated in the soma of the immunolabelled photoreceptor (Fig. 2). Depending on the species and retinal region, L cone densities ranged from 3,000/mm^2^ to 10,000/mm^2^ and S cone densities from <1,000/mm^2^ to 6,000/mm^2^. Overall, cones were more frequent in ventral than in dorsal retina. It is noteworthy that phyllostomid bats have a much higher percentage of genuine S cones (locally up to 60%) than other mammals including humans, where S cones commonly account for only about 10% of the cone population [2], [13]. An assessment of rod photoreceptors in the two phyllostomid species revealed rod densities of 130,000–390,000/mm^2^. Hence about 3% of all photoreceptors are cones. A very recent study of photoreceptors in the greater horseshoe bat (Rhinolophidae) reported similar rod and cone densities [10].

### Sequence Analysis of the S opsin

The spectral tuning of the S cone pigment was assessed by sequencing the tuning-relevant segment of the S opsin gene. The coding sequences of the S opsins of both species have been deposited in GenBank (accession numbers FJ815442 and FJ815443). In *C. perspicillata* and *G. soricina*, the tuning-relevant amino acids were identical to those of the mouse, shown to tune the mouse S opsin to UV rather than blue light [14] (Table 1). This strongly suggests that the two bat species also possess a UV-sensitive S cone pigment.

**Table 1:** Tuning-relevant amino acids of the mammalian S cone opsin.

Order	Species	λmax (nm)	52	86	93	114	118
Chiroptera	G. soricina*	≤365	Thr	Phe	Thr	Ala	Ser
	C. perspicillata*	≤365	Thr	Phe	Thr	Ala	Ser
	*Myotis velifer* [9]	–	Thr	Phe	Thr	Ala	Ser
	*Haplonycteris fischeri* [9]	–	Thr	Phe	Thr	Ala	Ser
	*Pteropus dasymallus* [9]	–	Thr	Phe	Thr	Ala	Ser
Rodentia	*Mus musculus* [33]	359	Thr	Phe	Thr	Ala	Ser
Insectivora	*Talpa europaea* [4]	–	Thr	Phe	Thr	Ala	Ser
Marsupialia	*Tarsipes rostratus* [34]	363	Thr	Phe	Thr	Gly	Ser
Primates	*Homo sapiens* [35]	424	Phe	Leu	Pro	Gly	Thr
Artiodactyla	*Bos Taurus* [35]	438	Thr	Tyr	Ile	Ala	Cys

Tuning-relevant amino acids of the mammalian S opsins in selected species with blue or UV sensitivity. *Mus musculus* and *Tarsipes rostratus* are species with known UV tuning (tuning wavelengths given in 3rd column). Data sources: *present study, [4], [9], [33]–[35].

### Adaptation and Sensitivity Range of the Phyllostomid ERG

**Figure 3:**
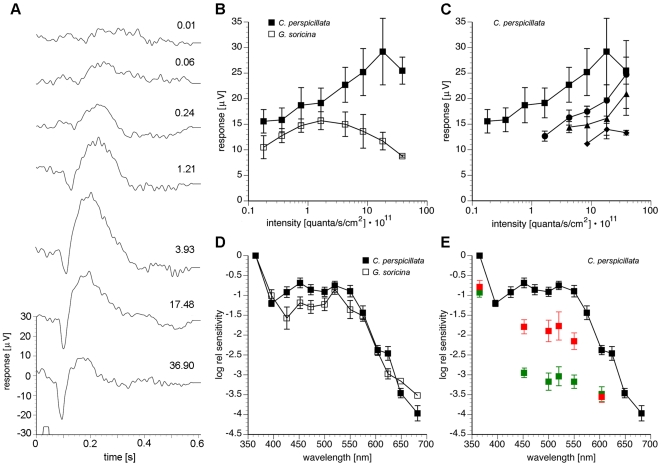
ERG responses of *C. perspicillata* and *G. soricina* at mesopic conditions. (A) Sample ERG responses from *C. perspicillata* to 550 nm light stimuli of increasing intensity (stimulus indicated on abscissa; duration 200 ms, stimulus intensities indicated near the traces, multiplied by 10^11^ quanta•s^−1^•cm^−2^). Each trace shows the average of 30–60 responses and is shifted vertically for clarity. (B) Intensity-response curves for 500 nm test flashes of increasing intensity in *C. perspicillata* (filled squares) and *G. soricina* (open squares). The peak response in *G. soricina* occurs at an approximately 10-fold lower intensity than in *C. perspicillata*. (C) Light adaptation in *C. perspicillata* was tested with 551 nm background illuminations of different intensities [0.18 • 10^11^ quanta•s^−1^•cm^−2^ (circles), 0.79 • 10^11^ quanta•s^−1^•cm^−2^ (triangles), 3.6 • 10^11^ quanta•s^−1^•cm^−2^ (diamonds)]. 500 nm test flashes of increasing intensity were presented. With increasing background illumination, the response to a given flash intensity decreases. Squares represent the situation with no adapting light (same curve as in B). Data points in (B) and (C) show mean±s.e.m.; n = 6 for *C. perspicillata* and n = 3 for *G. soricina*. (D) Cone contributions to the ERG were determined using spectral stimuli to obtain the action spectra S(λ) for *C. perspicillata* (filled black squares) and *G. soricina* (open black squares). Sensitivities were measured at 13 wavelengths (λ) ranging from 365 nm to 682 nm. Flash sensitivity at each wavelength was determined from the intensity required to reach a b-wave criterion response of 15 µV and normalized to 0 at 365 nm. (E) For *C. perspicillata*, action spectra were also measured during chromatic adaptation to background lights of 551 nm (filled green squares; 3.6 • 10^11^ quanta•s^−1^•cm^−2^) or 656 nm (filled red squares; 28.1 • 10^13^ quanta•s^−1^•cm^−2^) to assess the λ_max_ of the UV-sensitive pigment. The bleaching effect of the green background was stronger than that of the red background at intermediate wavelengths (450–550 nm), demonstrating rod-specific bleaching in this part of the spectrum. At the long- and the short-wave ends of the spectrum the effect of the green background was reduced in comparison to the effect of the red background. This indicates contribution of a UV and a long-wave cone photopigment. Test flashes in the chromatic adaptation measurements were 365 nm, 452 nm, 500 nm, 520 nm, 551 nm, 604 nm, and 649 nm. Data points in (D) and (E) show mean±s.e.m.; n = 9 for *C. perspicillata* and n = 8 for *G. soricina*. Absolute sensitivity at 365 nm was 5.84•10^−10^ 1(/quanta•s^−1^•cm^−2^) for *C. perspicillata* and 1.99•10^−10^ 1/(quanta•s^−1^•cm^−2^) for *G. soricina*.

To assess the functional properties of the observed photoreceptor arrangement, we recorded corneal electroretinograms (ERGs) in *C. perspicillata* and *G. soricina*. ERGs were measured under mesopic conditions (see Methods) to observe both rod and cone contributions. The maximal b-wave amplitudes of the investigated species were quite small (15–30 µV, Fig. 3A). The intensity-response function of the corneal ERG b-wave was determined using 500 nm test lights of increasing intensity at different light adaptation levels. We found that in *C. perspicillata*, b-wave saturation occurred at approximately tenfold higher light intensities than in *G. soricina* (Fig. 3B). Weak light adaptation considerably reduced the ERG responses (Fig. 3C). Under fully light-adapted (photopic) conditions, no ERG responses were detectable, suggesting that the sensitivity range of the bat retina is shifted to lower light levels than observed, for example, in the mouse [15], [16].

### Action Spectrum S(λ) of the Phyllostomid ERG

Cone contributions to the ERG were determined using spectral stimuli to obtain the action spectra S(λ). The stimulus intensity required to reach a 15 µV criterion response at each wavelength was used to calculate relative S(λ) functions (Fig. 3D). In both *C. perspicillata* and *G. soricina*, the S(λ) function was a trimodal curve, showing a major maximum at or below 365 nm (UV), and two smaller maxima at 450 nm (blue) and 520 nm (green). Both S(λ) curves differed distinctly from the mammalian rod action spectrum [17], indicating the additional contribution of cones to the ERG.

In *C. perspicillata*, intense monochromatic 551 nm (green) or 656 nm (red) background illumination caused a marked drop in the relative sensitivities in the S(λ) curve for wavelengths >400 nm (Fig. 3E). The bleaching effect of the green background was stronger than that of the red background at intermediate wavelengths (450–550 nm), demonstrating rod-specific bleaching in this part of the spectrum. On the other hand, the effect of the green background was reduced at the long- and short-range ends of the spectrum, compared to the effect of the red background. This clearly indicates the contribution of additional cone photopigments to the ERG.

Sensitivity of the ERG response was highest in the UV part of the spectrum, and red and green background illumination were least effective in the UV part. This indicates a UV-sensitive S cone mechanism. The slight reduction of the UV response observed with the red and green background argues for an additional contribution of the β-band excitation of all photopigments (rod opsin, L and S cone opsins) to UV sensitivity. The two smaller maxima of the S(λ) curves persist during chromatic adaptation.

### Transmittance of Ocular Media

**Figure 4:**
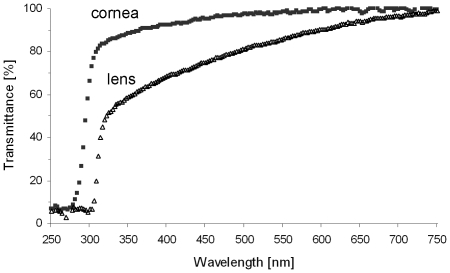
Spectral transmittance of cornea and lens of *C. perspicillata* from 250 to 750 nm. Mean values for four corneas and two lenses of two adult individuals are shown. Both cornea and lens showed high transmittance in the UV-range (310–380 nm) of the spectrum. Transmittance of the cornea was <10% below 280 nm but rose sharply to more than 80% transmittance at 300 nm and up to 100% towards 750 nm. The lens showed a sharp rise from <10% transmittance at 300 nm to 50% at 310 nm, and then a continuous increase to 100% transmittance at 750 nm.

In addition to UV-sensitive photoreceptors, UV-transmissive ocular media (cornea, lens, vitreous) are an essential prerequisite for detecting UV light. Therefore we measured the transmittance of both cornea and lens of *G. soricina* and *C. perspicillata* (Fig. 4) and showed that UV light (around 350 nm) in fact reaches the bat retina.

## Discussion

Our results demonstrate that phyllostomid bats have a significant cone population and thus conform to the common mammalian retinal blueprint [2], [13]. We assume that for bats the cones are most useful in mesopic (rod- and cone-stimulating) light conditions. The two cone pigments provide the basis for spectral contrast detection and perhaps true dichromatic colour vision. Our combined evidence shows that the spectral range extends into the UV.

### Spectral Sensitivity of Phyllostomid Bats

The action spectra obtained by corneal electroretinographic (ERG) recordings revealed, for the first time, a UV-sensitive cone pigment in *C. perspicillata* that is not affected by long-wave bleaching light. The action spectra corroborate our molecular evidence for a UV-tuned short-wave-sensitive (S) cone pigment. We conclude that the elevated sensitivity of the retina to UV light, in both species investigated, is attributed to the considerable proportion of cone photoreceptors exclusively expressing S opsin and the large number of cones co-expressing S and L opsins. Similar results were reported for mouse retina, which also has a high proportion of dual-pigment cones [18]. The only published electrophysiological study on spectral sensitivity of bat photoreceptors examined four microchiropteran species, including *Carollia perspicillata* did not address UV-sensitivity because stimuli were limited to wavelengths >440 nm [12].

Our ERG recordings provide no details on the contribution of the long-wave-sensitive (L) cone pigment, which we identified using immunocytochemistry and *in situ* hybridisation. The action spectrum (Fig. 3D) only allows us to postulate that the λ_max_ of the L pigment lies between 530 and 560 nm. This corresponds to the λ_max_ of 558 nm postulated for the L opsin in *Myotis velifer* on the basis of amino acid analysis [9]. The estimate of an L cone mechanism with peak absorption at 580 nm by Hope and Bhatnagar [12] is somewhat higher. Both values are rather long-wave shifted for a mammalian L opsin, particularly for species with a UV-tuned S opsin. The general pattern is a relatively fixed wavelength separation of the L and S opsins. Rodents with UV-tuned S opsins (λ_max_ around 365 nm) have L opsins tuned to approximately 510 nm; carnivores and artiodactyls with S opsins tuned to blue (λ_max_ around 440 nm) have L opsins tuned to approximately 555 nm [1]. The origin of the minor maximum of the S(λ) curve in the blue region (around 450 nm) of *G. soricina* and *C. perspicillata* remains enigmatic and needs further investigation. This minor maximum was also observed in the S(λ) curve of *Eptesicus fuscus* [12].

**Figure 5:**
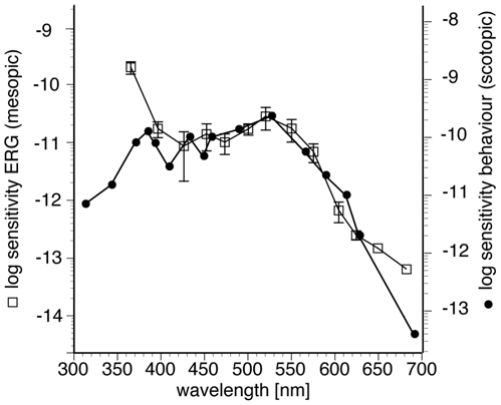
Comparison of action spectra for *G. soricina* obtained by different methods. Our ERG measurements (left ordinate, squares) were performed at mesopic light conditions, whereas behaviour data (right ordinate, circles) were collected under scotopic light conditions [11]. Absolute sensitivity is plotted against wavelength. For better comparison, the two sensitivity curves are shifted vertically to overlap at 520 nm; the behavioural response is actually about 1 log unit more sensitive (see discussion). The most noticeable difference is a higher UV sensitivity in the mesopic ERG curve. Sensitivities are given in 1/(quanta•s^−1^•cm^−2^).

Comparison of the action spectra S(λ) obtained from our ERG measurements and those obtained in behavioural experiments in dark-adapted *G. soricina* [11] show a good match between approximately 400 and 620 nm (Fig. 5). Relative sensitivity, however, was higher at 360 and 680 nm in our ERG measurements, indicating additional cone contributions in the UV and red regions of the spectrum. This difference is particularly evident in the UV range, supporting the presence of a UV-tuned S cone pigment. The elevated long-wave sensitivity could represent the long-wave tail of the L opsin tuning curve. The difference in absolute sensitivity of approximately one log unit between the ERG measurements and the behavioural experiments is attributable to the high criterion response chosen for the ERG measurements. Since the behavioural data were obtained under scotopic conditions [11], we assume that they reflect a pure rod sensitivity curve. This may also explain why the behavioural study did not observe colour discrimination in *G. soricina*. On the other hand, colour vision in bats may be less developed than in other mammals, even under cone-stimulating conditions.

### Biological Relevance of Cone-Based Vision for Bats

The present ERG data indicate that bat cones contribute to vision at mesopic light levels but become increasingly saturated at photopic light levels where mammalian cones usually operate. Mesopic vision at dusk and dawn and on brightly moonlit nights is particularly relevant for bats, since many bat species use visual cues for orientation and navigation between their daytime roosts and their feeding grounds [8], [19]. During foraging and homing, vision also plays an important role in predator avoidance, and, in some species, prey detection [20], [21]. Depending on their roosting situations, bats are exposed to different levels of ambient light during the day [22]. The different sensitivities observed in *C. perspicillata* and *G. soricina* (compare Fig. 3B) correlate with the respective roosting ecologies [22], [23]: *C. perspicillata* roosts in exposed locations, such as well-lit caves, hollow trees or under exposed tree roots, whereas *G. soricina* roosts in dim-lit caves.

### UV Vision in Mammals

With the present results, the Microchiroptera can be added to the mammalian taxa that have retained the ancestral UV tuning of their S cone pigment. So far, these include a number of rodents (most, but not all, nocturnal) [3], [24]–[27], a subterranean insectivore [4] and two marsupials [28]. It is unclear whether UV vision provides an adaptive advantage to these species, or whether there was simply no adaptive pressure on small-eyed nocturnal mammals to shift the S opsin tuning from UV to violet/blue. For diurnal mammals, the potentially damaging daylight UV levels are discussed as one factor driving evolution of UV-blocking eye media and blue-shifted S opsins [7]. Because of these UV-blocking optics, the opsin β-band–the secondary absorption peak in the UV region that is a property of the protein moiety of every visual pigment–cannot play a role in the vision of these animals. This is not the case in *G. soricina*, where we showed that both cornea and lens are transmissive for UV light, and a behavioural study under scotopic conditions [11] showed a contribution of the rod opsin β-band to vision. Under rod- and cone-stimulating (mesopic) light conditions, the UV sensitivities of the S opsin-containing cones and the rod and L cone opsin β-bands could combine to enable detection of UV-reflecting flowers [11]: some bat-pollinated neotropical plant species have violet blossoms and reflect UV light to a remarkable degree [29]. Interestingly, ambient light at dawn and dusk contains a particularly high proportion of short wavelengths [30]. Further, colour vision may also play a role in intraspecific communication, since some microbat species have distinct colour markings [22]. Further behavioural experiments at mesopic ambient light levels are needed to clarify whether bats make use of the two cone pigments for actual colour discrimination.

## Materials and Methods

### Animals

The study examined adult individuals of the phyllostomid bat species *Carollia perspicillata* (n = 24) and *Glossophaga soricina* (n = 16). Animals came from breeding colonies at Friedrich-Alexander University Erlangen, Germany; Goethe University Frankfurt/Main, Germany; and Ludwig-Maximilians University Munich, Germany.

### Ethics Statement

All procedures for animal handling, killing, and electroretinogram recordings complied with the NIH Principles of Laboratory Animal Care (NIH publication No. 86–23, revised 1996) and the corresponding German laws. A respective animal experimentation permit was granted by the Bezirksregierung Weser-Ems (Oldenburg), Germany.

### Immunocytochemistry and Photoreceptor Counts

Cone and rod immunolabelling and photoreceptor counts were carried out as previously described [31]. To label the visual pigments of cone and rod photoreceptors, we used antisera sc-14363 (Santa Cruz Biotechnology Inc., Santa Cruz, CA, USA) and JH455 against the S opsin, JH492 against the L opsin (both kindly provided by J. Nathans), and rho4D2 against the rod opsin (kindly provided by R. S. Molday). Primary antibodies were visualized by Alexa488- or Cy5-coupled donkey IgG secondary antibodies or by the peroxidase-anti-peroxidase (PAP) method. All variations of the staining protocol gave the same results. Photoreceptor densities were assessed in flattened whole retinae (c.f. Fig. 1D–F).

### In Situ Hybridisation

For localization of S and L opsin protein transcripts in the cone photoreceptor somata of *C. perspicillata*, we used in situ hybridisation following a published protocol [32]. In brief, whole retinae were prehybridised for 2 h at 60°C. Hybridization was carried out for 16 hours at 60°C in fresh hybridization buffer with the addition of denatured DIG-labelled riboprobes to antisense or sense mouse Opn1sw (50 ng/ml) or mouse Opn1mw (50 ng/ml). Riboprobes were generated by in vitro transcription of a T7 promoter-coupled PCR template (Opn1sw: nt 630–973, NM_007538; Opn1mw: nt 317–850, NM_008106) using T7 RNA polymerase and DIG-labelled rUTP (DIG RNA Labeling Kit, Roche). After washing according to the in situ hybridisation protocol, the colour reaction was carried out at RT and stopped after 8 hours. Retinae were rinsed in PBS and labelled with S and L opsin antibodies.

### S Opsin Sequencing

cDNA was synthesized from retinal RNA. Genomic DNA was extracted from muscle tissue. Primers 5′-GGA TGG GCC TCA GTA CCA C-3′ and 5′-GCA GTA GAT GAT GGG ATT GTA GAC-3′ were used for PCR amplification of the S opsin gene from exon 1 to exon 4. Reactions were conducted in 20 µl volumes on a MJ Mini Thermal Cycler (Bio-Rad, Hercules, CA, USA) with initial denaturation at 94°C for 3 min, denaturation at 94°C for 30 s, annealing at 57°C for 60 s, extension at 72°C for 90 s, for 35 cycles, followed by a final extension at 72°C for 5 min. Single products were obtained, amplified, purified, and directly sequenced on both strands.

### Electroretinographic (ERG) Recordings

For measuring the cone contribution to the bat ERG, we initially followed procedures used for measuring cone ERGs in other species by applying a rod-saturating, constant white light background illumination. It turned out, however, that even with relatively low-intensity white background illumination, no ERG responses were detected. Therefore, we subsequently worked at low mesopic conditions, where sufficiently large responses could be recorded, without strictly keeping the retina under scotopic conditions, in order to see the cone contributions.

*C. perspicillata* and *G. soricina* were adjusted to 12/12 hour light/dark cycles. ERG recordings were commenced shortly after the end of their resting periods in darkness, corresponding to starlight on a moonless night (0.03 lx; measured with a calibrated luxmeter; Palux, Gossen, Nürnberg, Germany). Animals were anesthetized by subcutaneous injections of xylazine (4 mg/100 g body weight) and ketamine (1.0 mg/100 g body weight), and the pupils were dilated with 1% atropine sulfate. Animals were then placed sideways on a preheated platform, fixed with tape and covered with preheated gel containers. Surgery and subsequent handling were done under dim illumination with red LEDs (corneal illuminance ranging from 5–10 lx).

A metal coil was placed around the eyeball using a manipulator, stabilizing it and keeping the eyelids open. A thin gold fibre electrode was then placed on the corneal surface, which was protected with a thin layer of Methocel. A platinum needle reference electrode was inserted subcutaneously into the skin covering the skull. Another platinum needle grounding electrode was inserted into the tail skin. Electrical potentials were recorded and band-pass filtered (1 to 1000 Hz) using a DAM 50 extracellular amplifier (WPI, Sarasota, FL, USA) connected to a PowerLab system (AD Instruments, Hastings, UK) for digitizing and storage.

The photostimulation system consisted of two light beams. The test light beam was used to obtain narrow-band test stimuli covering the range from 365 to 682 nm and originated from a 150 W xenon arc lamp. The image of the arc was focused onto the cornea using quartz lenses. The spectral content and intensity of this beam were controlled by sets of narrow-band interference filters (Schott, Jena, Germany; 6–13 nm half bandwidth) and neutral density filters with extended range in the UV (Zeiss, Oberkochen, Germany). The second beam served for continuous chromatic adaptation and originated from a 100 W halogen lamp. Its spectral content and intensity were also determined by narrow-band interference and neutral density filters. The light from this beam was focused onto one end of a glass fibre light guide. The other end completely illuminated the eye of the bat. The intensities of the test and adapting lights were measured in quanta•s^−1^•cm^−2^ at each wavelength with a calibrated, UV-enhanced photodiode (Oriel, 7182, Stratford, CT, USA) at the position of the cornea. Care was taken to correct for variation in the transmission of the neutral density filters at different wavelengths. The possibility that the UV filters transmitted a small fraction of long wavelength light sufficient to excite L cones was tested in a few experiments by adding a low-pass filter that transmitted only wavelengths longer than 450 nm. With this combination of UV narrow-band filter and low-pass filter, no responses were observed, indicating that the responses elicited by the UV light stimuli represented a true enhanced sensitivity in this region of the spectrum.

Even with the stabilized eyeball, the high breathing frequency of the bats introduced considerable variations in the baseline of the ERG recordings. Therefore, and because of the relatively small ERG responses, 30 to 60 light responses had to be averaged for each test stimulus. Stimulus frequencies of 1 Hz were used in order to finish measurements within 30 to 40 minutes, before anaesthesia started to degrade.

For analysis, a-wave response amplitudes were measured relative to baseline, which was determined by the mean voltage within a 50 ms period before the light flash. B-wave amplitude was determined from the most negative a-wave trough to the b-wave peak. Recording and analysis were performed with SCOPE v 4 and CHART v 5, respectively (AD Instruments, Hastings, UK). Statistical analysis was done with JMP 5.0 (SPSS Inc.), and results were plotted using Deltagraph v 5 (SPSS Inc).

Several independent measurements were performed with *C. perspicillata*. Intensity-response curves were measured with 500 nm or 550 nm test flashes of increasing intensity. The effect of light adaptation was tested with 500 nm or 550 nm test flashes of increasing intensities and 551 nm or 656 nm adapting light of different intensities, in order to estimate the background intensities suitable for chromatic adaptation. For the same reason, some experiments with the same adapting wavelengths were performed with a broadband UV filter (UG1, Zeiss, Oberkochen, Germany) in the test light beam (not shown). Finally, action spectra were constructed from sensitivity measurements at different wavelengths ranging from 365 to 682 nm. The flash sensitivity at each wavelength was determined from the intensities needed to reach a b-wave criterion response of 15 µV. In the same experiments, action spectra during chromatic adaptation to 551 nm or 656 nm background light were measured for test flashes of 365 nm, 452 nm, 500 nm, 520 nm, 550 nm, 604 nm, and 649 nm. In the case of *G. soricina*, only intensity-response curves and action spectra without (chromatic) adaptation could be measured, since it turned out that even small amounts of constant adapting light suppressed the small ERG responses below the noise level of the recordings.

Due to the red preparation light, the retinae were not completely dark adapted, but probably in a mesopic state. In addition, the relatively high stimulation frequency probably led to some additional light adaptation during the intensity-response measurements. This is clearly visible in the V – log I curve of *G. soricina*, where the amplitude severely decreased at higher light intensities (see Fig. 3B). *G. soricina* seemed to be more susceptible to light adaptation than *C. perspicillata*, since it was also not possible to obtain sufficiently large light responses with weak chromatic adaptation. In *C. perspicillata*, light adaptation was less severe, as evident from the prominent a-wave even at high light intensity stimulation in the recordings and the V – log I curves (Fig. 3A–C). In order to reach the state where a “mixed” ERG response was obtained (rod and cone contributions to the b-wave), a rather high criterion response was chosen for the action spectra measurements, reaching 50% of the maximal response in the case of *C. perspicillata*, and nearly 100% in the case of *G. soricina*. This is clearly not in the linear range of the V – log I curve, as usually desirable. On the other hand, however, this high criterion response (and the mesopic state) ensured that cone contribution was visible in the ERG responses, as evident from the action spectra (Fig. 3D&E).

### Lens and Cornea Transmission

Transmission of lens and cornea were recorded using a NanoDrop® ND-1000 UV-Vis spectrophotometer (NanoDrop Technologies, Wilmington, DE, USA). Four lenses and four corneas of two individuals of *C. perspicillata* and *G. soricina* were dissected, rinsed in 0.1 M PB, and clamped in a 0.2 mm light path between the fibre optics of the spectrophotometer. Measurements were made at 3 nm intervals from 250 to 750 nm.
